# A Practical Approach for a Wide Range of Liver Iron Quantitation Using a Magnetic Resonance Imaging Technique

**DOI:** 10.1155/2012/207391

**Published:** 2012-12-11

**Authors:** Ping Hou, Uday R. Popat, Richard J. Lindsay, Edward F. Jackson, Haesun Choi

**Affiliations:** ^1^Department of Imaging Physics, The University of Texas MD Anderson Cancer Center, 1515 Holcombe Boulevard, Houston, TX 77030, USA; ^2^Department of Stem Cell Transplantation and Cellular Therapy, The University of Texas MD Anderson Cancer Center, Houston, TX 77030, USA; ^3^Department of Diagnostic Radiology, The University of Texas MD Anderson Cancer Center, Houston, TX 77030, USA

## Abstract

The goal of this study is to demonstrate a practical magnetic resonance imaging technique for quantifying a wide range of hepatic iron concentration (HIC) for hematologic oncology patients with transfusion iron overload in a routine clinical setting. To cover a wide range of *T*_2_* values from hematologic patients, we used a dual-acquisition method with two clinically available acquisition protocols on a 1.5T MRI scanner with different ΔTEs to acquire data in two breath-holds. An in-house image postprocessing software tool was developed to generate *T*_2_*, iron maps, and water and fat images, when fat is presented in the liver. The resulting iron maps in DICOM format are transferred to the institutional electronic medical record system for review by radiologists. The measured liver *T*_2_* values for 28 patients ranged from 0.56 ± 0.13 to 25.0 ± 2.1 milliseconds. These *T*_2_* values corresponded to HIC values ranging from 1.2 ± 0.1 mg/g to 45.0 ± 10.0 mg/g (dry weight). A moderate correlation between overall serum ferritin levels and *R*_2_* was found with a correlation coefficient of 0.83. Repeated phantom scans confirmed that the precision of this method is better than 4% for *T*_2_* measurements. The dual- acquisition method also improved the ability to quantify HIC of the patients with hepatic steatosis.

## 1. Introduction 

Patients with transfusion-dependent hematologic malignancies, such as myelogenous leukemia, can often be treated successfully with stem cell transplantation [1–3]. However, after stem cell transplantation, transfusional hemosiderosis may develop. If left untreated, the cumulative effects of iron overload may lead to significant morbidity or even mortality. The liver is the first and foremost organ affected by iron overload, and hepatic iron overload has been associated with the development of hepatitis, hepatic fibrosis, and cirrhosis. The heart can also be affected by iron overload, resulting in heart failure. Thus, it is important to accurately diagnose and adequately treat iron overload, especially in patients with transfusion-dependent hematologic malignancies. 

Since much of the body's excess iron is deposited in the liver, hepatic iron concentration (HIC) is often used as a surrogate for the total body iron load. Liver biopsy is currently used to confirm the diagnosis of the iron overload disease and to monitor it during the course of treatment. However, liver biopsy is an invasive procedure with significant risk in patients with low platelet counts, and the technique to quantitate iron in liver tissue is not widely available. Because of the limitations of liver biopsy, a noninvasive method of hepatic iron detection and quantification, such as MRI, has been investigated by many researchers [4–24]. It has been shown that the transverse relaxation times *T*_2_ and *T*_2_* are inversely proportional to HIC [5], which means that the transverse relaxation rates *R*_2_ and *R*_2_*, which are the reciprocals of *T*_2_ and *T*_2_*, respectively, are directly proportional to HIC. Using various MRI techniques, several investigators have measured HIC from *T*_2_ decay curve by spin echo (SE) method [7, 8, 14] and *T*_2_* decay curves generated by gradient recalled echo (GRE) sequences [8–10, 20–25]. Another method widely used in Europe [11–18] is based on signal ratio of liver over reference tissue, using multiple images such as *T*_1_-,*T*_2_*-, proton-density- (PD-) weighted images from GRE and SE sequences. In general, *T*_2_ method takes longer time to scan, each breath hold can only generate one image at one echo time. *T*_2_* method is faster, more sensitive to iron concentration and generates multiple images at different echo time within one breath hold. In addition, Wood et al. [8] demonstrated that the *T*_2_ and *T*_2_* method generated comparable HIC value. The HIC values measured from MRI *R*_2_* maps have further been validated by other investigators against the HIC values obtained from the chemical analysis of needle biopsy specimens, which is considered the standard method for measuring HIC [8, 9]. Wood's method used only one protocol with relatively short TE (maximum TE = 4.8 msec) and short ΔTE (0.8 msec), which may result in greater uncertainty in long *T*_2_* data acquisition.

In oncologic patient, accumulation of fat in the liver following extensive chemotherapy is often a clinical concern. Using magnitude images acquired by multiecho gradient echo sequence (MEGE), several researchers have measured proton density fat fraction (PDFF) [26–28]. And they pointed out that *T*_2_* would complicate fat quantification. In all of these published studies, four to eight echoes are acquired, with each echo set at the time of water-fat in and out of phases so that fat fraction can be measured accurately. However, short *T*_2_* is not measured adequately because the minimum TE and ΔTE is not short enough to catch the fast decay MR signal from high iron overloaded liver. 

In clinical practice, we encounter patients with a wide range of HIC, from severe iron overload with very short *T*_2_* (<2 msec) images to moderate-low iron overload with relative long *T*_2_*. Currently, there is no “one size fits all” technique for liver iron quantification. The aim of this work is to (1) optimize clinically available MR acquisition protocols to cover a wide range of *T*_2_* decay; (2) develop postprocessing tools to generate reliable HIC maps; (3) generate and display the HIC map in a timely manner for radiologists to review. Our method, employing two MEGE acquisition protocols with different TE coverage to cover a wide range of *T*_2_*, is an extension of Wood's work. It not only measures wide range of clinically relevant *T*_2_*, but also allows us to simultaneously measure HIC and fat fraction for patients with hepatic steatosis. 

## 2. Materials and Methods

### 2.1. Patient Population

This retrospective study was performed with the approval of our institutional review board, which waived the requirement for informed consent. We identified 28 consecutive patients with high ferritin who underwent hematopoietic stem cell transplant at our institution. Of these 28 patients, 19 had acute myelogenous leukemia (AML), 3 had myelodysplastic disease (MD), 3 had chronic myelogenous leukemia (CML), and 3 had myelofibrosis. Clinically, it is believed that the amount of ferritin in blood reflects the amount of iron stored in the liver [13]. Therefore, the serum ferritin level was measured within one month of the MRI scan in all patients. 

### 2.2. MRI Techniques

All MRI scans were performed using an 8-channel Torso cardiac coil on a 1.5-T MRI scanner (General Electric Medical Systems, Milwaukee, WI, USA), with a maximum gradient of 40 mT/m and a slow rate of 150 mT/m/sec. After acquiring standard abdominal MR images, such as *T*_1_W in-out of phase and respiratory triggered *T*_2_W images, we acquired MEGE *T*_2_*-weighted images through the largest section of the liver in the axial plane away from major vessels. An oblique coronal was also acquired in order to check liver heterogeneity. The lung was excluded on the axial plane as much as possible to avoid introducing potential susceptibility artifacts.

From curve fitting point of view, we should acquire many echoes with short ΔTE (less than 1.0 msec) to cover the entire *T*_2_ decay curve so that *T*_2_* value could be accurately determined. The clinically available MEGE sequence can only acquire the maximum of sixteen echoes on our scanner, which limits the range of *T*_2_* that can be measured accurately if inadequate ΔTE (either too high or too low) is used. For a given patient, without prior knowledge of *T*_2_* or iron concentration, it is difficult to select the “right” ΔTE without “trial and error.” To overcome this difficulty, we used two MRI acquisition protocols with different ΔTEs for each patient to acquire data in two breath-holds. Of the two measurements, the one that had better correlation and smaller chi-square value was selected for subsequent *T*_2_* and iron map generation. 

The imaging parameters were optimized to include the following considerations: (1) the shortest TE and a reasonable ΔTE for curve fitting; (2) a high enough signal-to-noise ratio (SNR) to allow the liver to be differentiated from background noise for very fast *T*_2_* decay cases; (3) a short enough scan time to allow a single breath-hold for most patients. The long TE protocol was for long *T*_2_* (>3 msec) cases using a single shot, TR = 39.1 msec, 12 echoes, a unipolar readout gradient, TE1 = 1.448 msec, and ΔTE = 2.336 msec. The short TE protocol was for short *T*_2_* (<3 msec) cases using double shots, 8 echoes/shot, TR = 20 msec, a bipolar readout gradient, TE1 = 1.448 msec, and ΔTE = 0.636 msec. Each protocol had a scan time of less than 16 seconds, with the longest TEs being 27 msec and 11 msec, respectively. The other imaging parameters, including matrix size (256 × 192), field of view (38 cm), flip angle (25°), receiver bandwidth (125 kHz), slice thickness (10 mm), and averages (2), were kept the same in all protocols. Among the two acquisitions, only one of the *T*_2_* fitted images with the higher correlation coefficient R of the fit, was selected to generate HIC map for clinical use. 

To validate the reproducibility of our method and test the stability of the system, a FerriScan phantom was used to measure *T*_2_* values with the same protocol for patients in every three to four months over a period of one year. This phantom consists of 15 vials of aqueous manganese chloride (MnCl_2_) solutions (0 mM to 3.2 mM) in 10 mM hydrochloric acid, which provide the *T*_2_* values from 3.0 msec to 40.0 msec, similar to the range of liver *T*_2_* values for patients. It is the same type of phantom used by Pierre et al. [7], who validated their technique for HIC measurement using the phantom and biopsy data. Their single echo SE based technique would take 20–30 minutes to scan. 

### 2.3. Postprocessing MR Images 

The *T*_2_*, *R*_2_*, and hepatic iron maps were processed off-line using an in-house developed software tool based on MATLAB (Mathworks, Inc., Natick, MA, USA). The *T*_2_* curve was fitted to a monoexponential decay with three parameters using the Levenberg-Marquardt nonlinear least-squares method:

(1) $$\begin{array}{c} S\left(t\right)=A_{0}\exp\left(-\frac{t}{T_{2}^{*}}\right)+C, \end{array}$$

where *A*_0_ is the signal amplitude at time 0, with its initial fit value set to 1.5 or 5.0 times of the maximum signal of the first echo for each pixel for long *T*_2_* or short *T*_2_* cases; *T*_2_* is initially set to 10 msec; and *C* represents noise whose initial fit value was set to the background noise. The background noise was measured from a small ROI in the frequency direction; its mean was used as a threshold to roughly mask the image out before fitting. When there is fat in the liver, the MRI signal in the liver can be represented by

(2) $$\begin{array}{c} S\left(t\right)=\left(W+F\exp\left(i\omega t\right)\right)\exp\left(-\frac{t}{T_{2}^{*}}\right)+C, \end{array}$$

where *W* is the water signal amplitude, *F* is the fat signal amplitude, and *ω* is the water-fat frequency difference that is about 220 Hz at 1.5 T. Other parameters are the same as in (1).

After fitting *T*_2_* curve using (1) or (2), the iron value, Fe in mg/g (dry weight), was calculated based on a previously validated linear relationship between iron and *R*_2_* value in Hz [8]:

(3) $$\begin{array}{c} F_{e}=0.0254\cdot R_{2}^{*}+0.202. \end{array}$$

Both region of interest (ROI) and pixelwise fitting were implemented for a quick evaluation and *T*_2_* map generation. A small ROI was drawn to plot the signal intensity versus time curve for a quick review. If there was no signal oscillation, that means there was no fat in the liver, (1) was used for curve fit; otherwise (2) was used for curve fit. To reduce noise, the original images were smoothed by a 3 × 3 window kernel prior to curve fitting for short *T*_2_* cases. Since image intensity is not uniformly distributed due to liver heterogeneity, tissue susceptibility, and surface coil sensitivity, local ROI measurements cannot represent the global iron overload of the liver. After the pixelwise *T*_2_* curve fitting, we segmented out the liver from the entire image to form liver-only HIC map and generated a histogram of the liver-only HIC map with a normal distribution fit. This histogram shape, mean and standard deviations were used for longitudinal HIC comparison for patient followups.

The iron map generated by (3) was saved as a Digital Imaging and Communications in Medicine (DICOM) image, its DICOM header was derived from original MRI image with different description and series number, and was transferred to the institutional electronic medical record system, ClinicStation (Datalign, Inc., Houston, TX, USA), for measurement and interpretation by radiologists. Radiologists can obtain HIC values directly from the iron map by drawing an ROI.

## 3. Results

For all 28 patients, the average *T*_2_* values ranged from 0.56 ± 0.13 msec to 25.0 ± 2.1 msec. The corresponding HIC values calculated from (1)–(3) were from 1.2 ± 0.10 mg/g to 45.0 ± 10.0 mg/g dry weight. 

**Figure 1:**
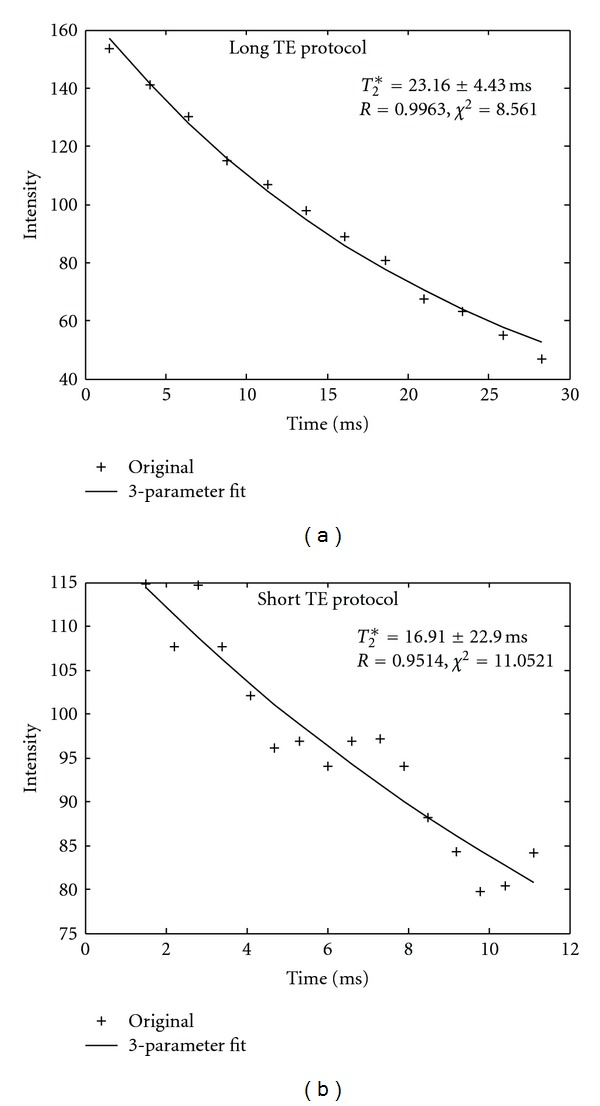
Results of *T*_2_* curve fit from a patient with long *T*_2_* values by dual acquisition method. (a). Data was acquired by the long TE protocol. (b) the same patient data was acquired by the short TE protocol. It is obvious for this case that the *T*_2_* fit error is increasing with shorter TE coverage. The long TE data acquisition is necessary for mild iron load patient.

**Figure 2:**
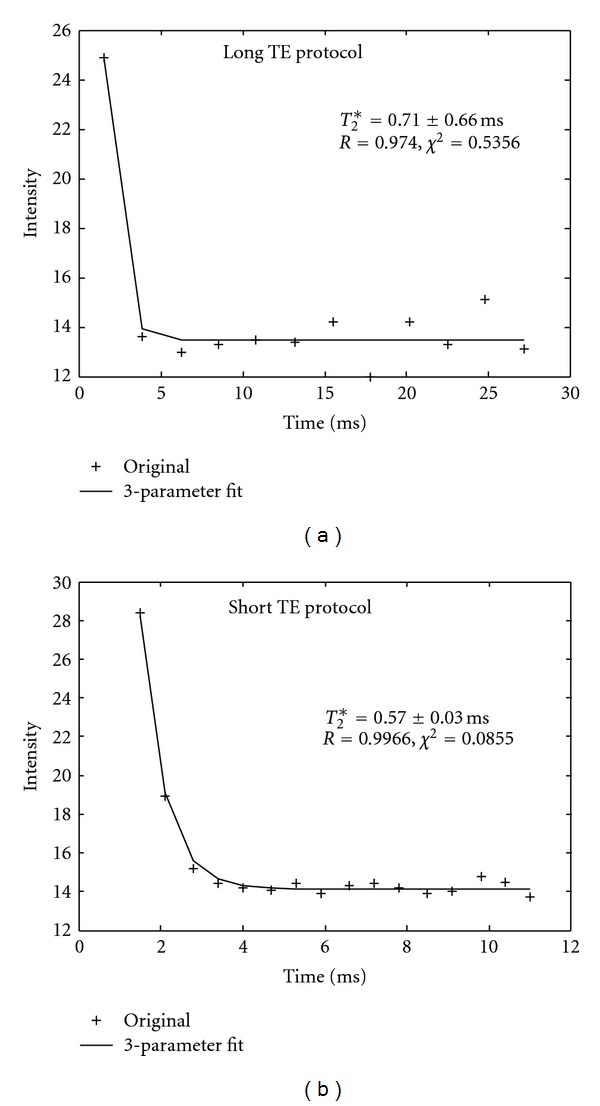
Results of *T*_2_* curve fit from a patient with short *T*_2_* values by dual acquisition method (Figure 1(a)). Data was acquired by the long TE protocol (Figure 1(b)). The same patient data was acquired by the short TE protocol, there were more data points in the curvature area. It is obvious that data acquired by short TE protocol generated better fit for short *T*_2_* case.

Figure 1 shows a representative fitted signal versus time curves with long *T*_2_* value. Dual data acquisition method was applied to the same patient, with fitted curves as shown in Figures 1(a) and 1(b), respectively. The *T*_2_* decay curve acquired from the long TE protocol resulted in the better fit for the long *T*_2_* case, as demonstrated in Figure 1(a). Therefore, the image from the long TE protocol was selected to generate an iron map.  Figure 2 is an example of a very short *T*_2_* case with dual acquisition method in (a) and (b), respectively. The data acquired by the short TE protocol (for short *T*_2_* values) provided better fit (b), as demonstrated by its correlation coefficient and chi-square value. Therefore, in this case, images acquired by the short TE protocol were used to generate an iron map. In general, the more points sampled near the part of the curve with larger curvature, the more reliable the generated results. For this reason, the longer TE and ΔTE spans were used for the long *T*_2_* fit, and the shorter TE and ΔTE spans were used for the short *T*_2_* fit. As shown in Figure 2, the short *T*_2_* had faster decay and generated very low-intensity liver images. The signal intensity after the first echo acquired by the long TE protocol, or after the third echo acquired by the short TE protocol, was very close to the background noise and thus was much weaker than the signal from the long *T*_2_* (Figure 1) case.

**Figure 3:**
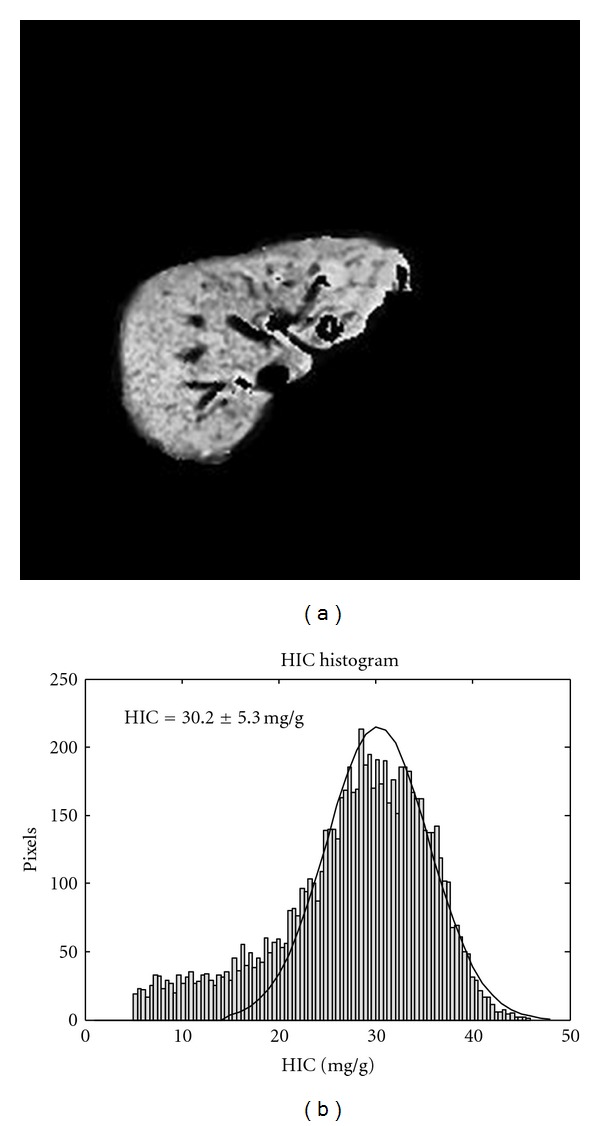
A 31-year-old man with acute myelogenous leukemia, sixteen months after bone marrow transplantation. (a) is the HIC map of the segmented liver. (b) is its corresponding histogram with normal distribution fit. This patient had very high iron deposition with mean HIC value of 30.20 mg/g (*T*_2_* < 1.0 msec).

Figure 3 displays a typical HIC map and its histogram of a 31-year-old man with acute myelogenous leukemia who had very high levels of iron deposition.  Figure 3(a) is the HIC map of the segmented liver, and the corresponding histogram with normal distribution fit is shown in Figure 3(b). A combination of the HIC map and liver-only histogram provide local and global measurements of the response of the liver to the treatment. 

**Figure 4:**
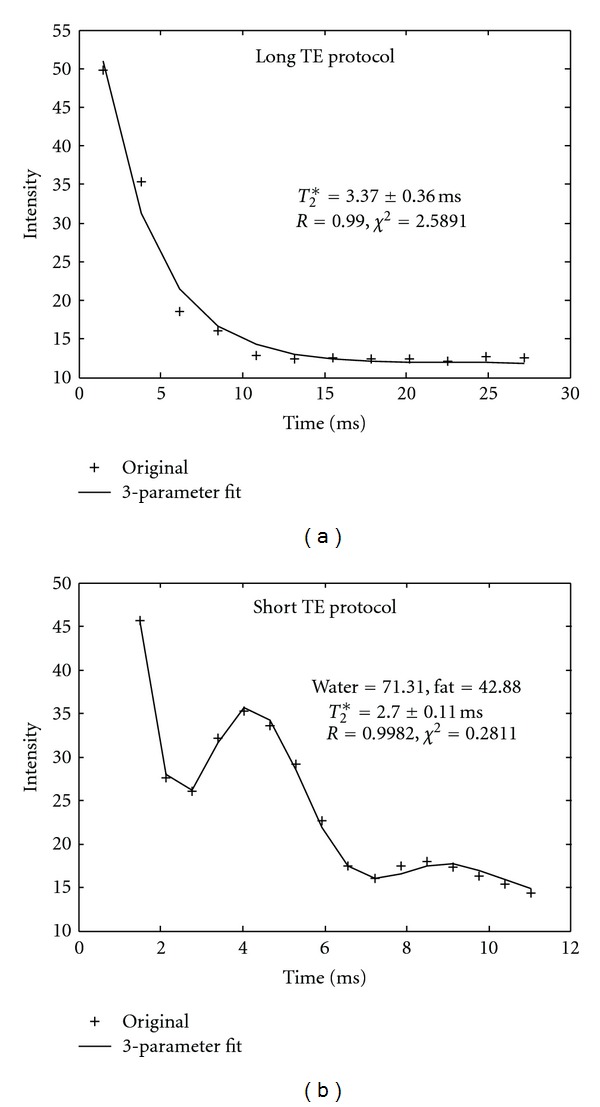
An example of *T*_2_* curve fit from a liver steatosis patient with short *T*_2_* values and by dual acquisition method. (a) Data was acquired by the long TE protocol. The water and fat peaks were totally missed because data was sampled too slowly. (b) The same patient data was acquired by the short TE protocol with faster sample (shorter ΔTE). Water and fat peaks were caught and data was fitted with water and fat components. Again data acquired by the short TE protocol generated better fit for short *T*_2_* case.

**Figure 5:**
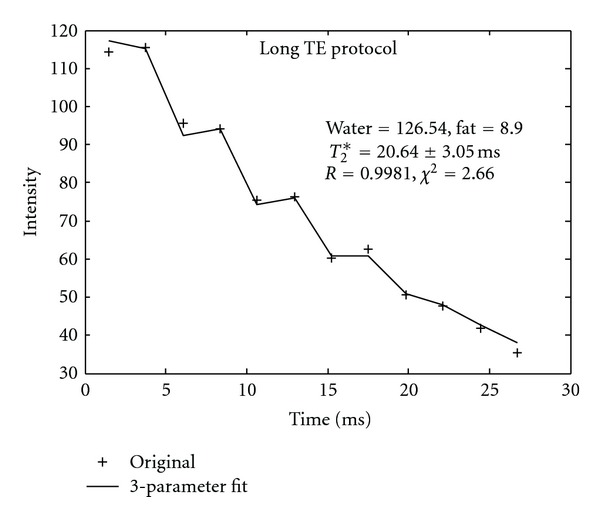
An example of *T*_2_* decay curve fit from a liver steatosis patient with less iron deposition. The long TE protocol was adequate to catch the water and fat peak while minimum fit error for long *T*_2_* decay was achieved.

Figure 4 is an example of *T*_2_* decay curves from a hepatic steatosis patient acquired by our dual acquisition method. There was no water-fat signal oscillation from the long TE acquisition protocol in Figure 4(a). However, water-fat peaks were clearly demonstrated with the data acquired by the short TE protocol, as shown in Figure 4(b).  Figure 5 demonstrates that the long TE acquisition protocol is necessary to measure mild iron concentration as well as PDFF for a hepatic steatosis patient.

**Figure 6:**
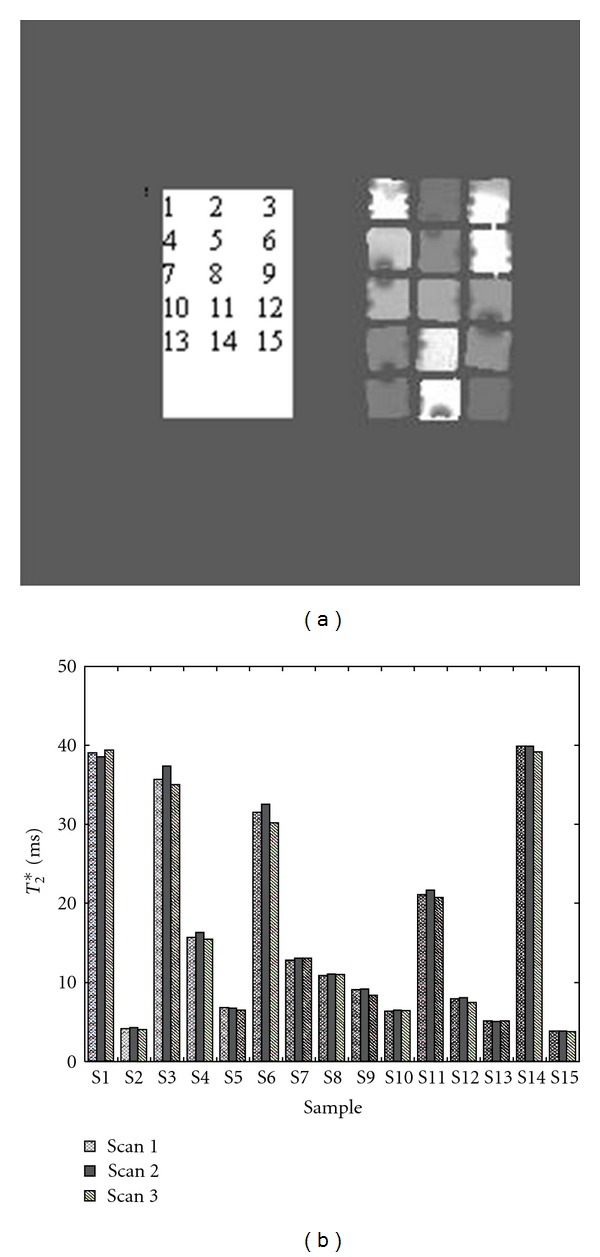
Results of phantom validation. (a) is the image of the FerriScan phantom consisting of fifteen different tubes with different solutions (different *T*_2_* values). The sample numbers are defined on the left. (b) is the *T*_2_* measurement from three scans in a year using the same clinical protocols. Consistent measurement was demonstrated in this plot.

Shown in Figure 6 are the *T*_2_* measurement results (b) from a FerriScan Phantom consisting of fifteen different liquid tubes (a) with different *T*_2_* values. Three data sets were acquired with the same protocol for patient scan in a year with 3-4 months apart. 

**Figure 7:**
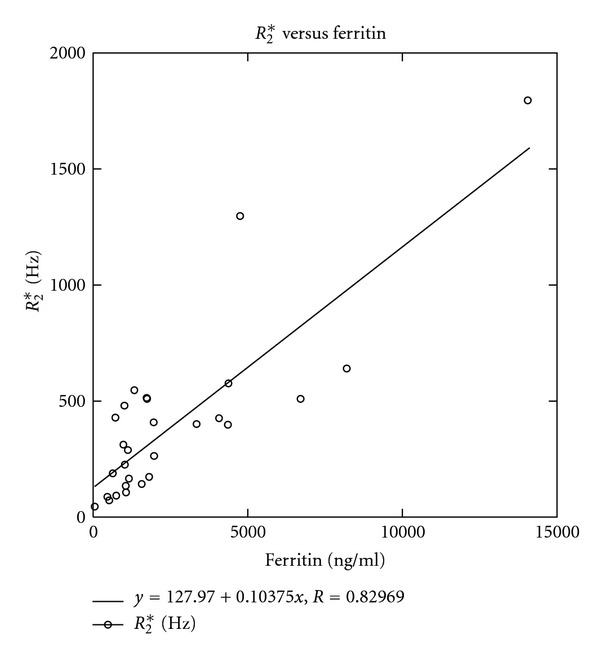
Scattergram of *R*_2_* values calculated from MEGE MRI acquisition versus serum ferritin values. A good correlation between *R*_2_* and ferritin is clearly demonstrated.

Figure 7 presents the relationship between *R*_2_* and ferritin for all patients. A paired Student's *t* test was run between *R*_2_* and ferritin values, with a correlation of 0.83 and *P* = 0.0001. 

## 4. Discussion

We have developed a simple and practical MRI strategy that provides the absolute HIC. By combining two different MEGE data acquisition protocols and an in-house image postprocessing tool, we cannot only measure a wide range of clinically relevant iron loads, from 1.2 mg/g to 45 mg/g (dry weight), but also quantify water and fat simultaneously with confidence. This technique is now routinely used to evaluate HIC in all patients with suspected iron deposition disease in our institution.

For patients with high levels of hepatic iron deposition (e.g., HIC > 15 mg/g), the *T*_2_* value was very short, and thus a short ΔTE data acquisition method should be used. For patients with low levels of hepatic iron deposition (e.g., HIC < 3 mg/g), the *T*_2_* value was long and thus a relative long ΔTE and longer TE coverage data acquisition method should be used. Inadequate data acquisition protocol would result in great uncertainties in the value of HIC, as demonstrated in Figures 1 and 2. In this study, because the MRI scans were performed without prior knowledge of each patient's HIC, dual acquisition method covering different ranges of TE span was used. Since each acquisition took less than 16 seconds, we were able to scan each patient with both protocols (for different ΔTEs covering the entire range of possible *T*_2_* values) in two breath-holds. The data acquired with the longer TE span generated better fits for the long *T*_2_* curve (Figure 1(a)), and the data acquired with the shorter TE span generated better fits for the short *T*_2_* curves (Figure 2(b)).

A popular method to quantify liver iron by MR in Europe is based on signal ratio of liver over reference tissue [11–18] from different images. Recently, this method has been further developed into a web-based iron measurement (URennes) [14] where proton-density-, *T*_1_-, *T*_2_-, and *T*_2_*-weighted images have to be acquired based on their protocol, and certain ROIs have to be measured in the liver and reference tissues as the inputs to calculate an adequate iron value. Gandon et al. [16] also validated this technique with biopsy data on 1.5T system, declaring that it could be used on various MR scanners. Most recently, Castiella et al. [18] reevaluated this method using the data from multiple institutions with different scanners in the period of 1999 to 2006. They investigated the accuracy of this method with liver biopsy and found that the diagnostic accuracy was 61.4% with a tendency of overestimate overload. They also found that the iron concentration by this method was less reliable from 60 *μ*mol/g to 170 *μ*mol/g, corresponding to 3.35 mg/g to 9.5 mg/g. Another limitation of this method was that it could only measure iron up to 350 *μ*mol/g (19.5 mg/g). This method was developed early in late 1990s [12] when MEGE sequence was not commercially available.

Wood et al. [8] studied more than 100 patients for HICs by *T*_2_ (SE), *T*_2_* (GRE) method. Among them, 21 were liver biopsied and validated. They reported that the GRE sequence was able to adequately measure *T*_2_* values in the entire range of iron overload. This method has recently been further validated by other investigators [9]. In our study, although the minimum TE was 1.448 msec, we were still able to measure *T*_2_* values less than 1.0 msec from 8-echo, double-shot interleaved sequences, as demonstrated in Figure 2(b), where the SNR dropped to the background level at the fourth echo. This *T*_2_* value corresponds to a HIC value over 40 mg/g for that specific ROI, which is extremely high for a patient with acute myeloid leukemia [7, 8]. For such high iron overload, raw images need to be smoothed to remove some noises. It is known that the *T*_2_* value for normal liver is ~24 msec [20–22]. Therefore, we have demonstrated that our technique is able to measure the entire range of possible clinical HIC values using the clinically available MRI sequence. With the maximum receiver bandwidth used in our protocol, the first TE could be reduced further by using a 128 × 128 or even 64 × 64 acquisition matrix. This might be beneficial for SNR for cases with very short *T*_2_* values (<1.0 msec). Meanwhile, Figure 1 demonstrates the importance of long TE acquisition for long *T*_2_* decay case. Short TE acquisition for long *T*_2_* decay case would result in larger measurement uncertainty, a limitation of Wood's method. 

The slope and intercept for the linear equation (3) between HIC and *R*_2_* under 1.5-T was determined by Wood et al. [8] using biopsy results. It was also calibrated and confirmed with biopsy values by Hankins et al. [9]. In our study, we used Wood et al.'s results and did not validate our HIC values against those from biopsies. In addition, Wood pointed out that their calibration was validated from 1.3 mg/g to 32.9 mg/g dry weight. There was an outlier with HIC value of 57.8 mg/g, it was removed from their statistic calibration. With the minimum TE of 1.448 msec in our dual acquisition method, although we can measure and fit the *T*_2_* value as short as 0.57 msec, as demonstrated in Figure 2(b), we did observe significant noise from data acquisition, especially close to the high tissue susceptibility area and low coil sensitivity area. For patients with high HIC values, having an HIC map is necessary to view the image nonuniformity, an example is shown in Figure 3(a). However, this HIC image map is not enough to track the overall mean HIC value and standard deviation. A liver-only HIC histogram with normal distribution fit, as shown in Figure 3(b), can provide the mean and standard deviations of the liver's iron concentration. We have found that a combination of HIC image map and its histogram distribution is extremely useful for patient followup after treatment.

Our dual acquisition method cannot only cover wide range of clinical relevant *T*_2_* decay (all iron deposition), but also quantify iron accurately for the patient with superimposed steatosis, as demonstrated in Figure 4. For hepatic steatosis patient with less iron deposition, the long TE protocol can detect water and fat oscillation, as shown in Figure 5. Meanwhile, the short TE protocol with shorter ΔTE can detect the water and fat oscillation for patient with high iron deposition. It is well known that water and fat will be in phase at time 4.6 msec and out of phase at 2.3 msec on 1.5 T system. By setting the echo time at the multiple time of 2.3 msec, Sirlin and Reeder [26–28] studied fat quantification by measuring PDFF using an MEGE sequence. Their methods can quantify fat and water, as well as generate *R*_2_* map with four to eight echoes. Their main purpose was fat assessment; therefore, the minimum TE and ΔTE were not short enough to quantify high iron overload cases. For extremely high iron overload such as *T*_2_* < 1.0 msec, steatosis could not be detected by image technique because water and fat peak may not be MRI visible. In our study, no fatty liver was detected with *T*_2_* value less than 2.0 msec. To improve the accuracy of HIC and fat measurement, a better pulse sequence is needed with the minimum TE and ΔTE less than 1.0 msec, the maximum TE greater than 20.0 msec, and some of the echo should be set at multiple times of 2.3 msec.

Chebrolu et al. [29] pointed out that the independent *T*_2_* decay for water and fat model may improve accuracy of water-fat quantification using a 3D MEGE sequence, particularly for high fat fraction and short *T*_2_* case. We have used independent *T*_2_* model to fit the signal decay curve. For our limited number of steatosis patients, we have not observed curve fit improvement. Therefore, in our study, we assumed that the *T*_2_* decay was identical for water and fat signal (2). The independent *T*_2_* decay model needs to be further studied with a larger number of patients. 

Researchers [21, 22, 30, 31] have demonstrated that iron measurement from *T*_2_* method has high reproducibility and inter-MRI scanner agreement. Using FerriScan phantom, we have confirmed the reproducibility (Figure 6) of *T*_2_* measurements for all samples. The average error was 2.1% with the maximum error of 3.9%. The largest error mainly came from nonuniform image due to phantom/slice position between each scans, seen in sample 3 and 6. The consistent measurement from fixed phantom samples further demonstrated that our technique is quite promising for patient followups. In our study, we have found that consistency in data acquisition and analysis is vital for patient followups.

As mentioned earlier, there was a reasonably good correlation between the *R*_2_* and ferritin values (Figure 7) in our study. Although the serum ferritin values have been used as a marker for body iron amount [19], some researchers [9, 10] have shown that the ferritin values do not necessarily reflect the actual total body iron burden. Therefore, using serum ferritin to represent iron load is still a debatable topic. On the other hand, HIC as measured by an MEGE sequence does represent the actual hepatic iron burden, as supported by several biopsy reports [7–9]. Using the dual acquisition method described in this study, the MEGE sequence can be used in the clinical decision-making with higher confidence. 

Image quality will affect the iron measurements. First, susceptibility artifacts will affect our measurement of HIC. Shimming could help to reduce some B0 inhomogeneity, and positioning the patient carefully so that liver is not close to the edge of coil will help to generate more uniform image. Since the whole HIC map is generated, radiologist can always put an ROI to the most homogeneous part to measure HIC value. The histogram from liver-only HIC provides overall mean and standard deviations, a useful distribution to monitor treatment. Meloni et al. [25] have studied single-slice versus multislice HIC distribution of HIC by *T*_2_* method and concluded that the single-slice measurement is enough in the clinical application as long as major vessels are avoided. Second, patient's motion affects the *T*_2_* measurement, breathing resulting in severe nonuniform artifact in the liver image. Breath instruction should be delivered clearly before data acquisition and patients need to be trained to hold breath. 

Our technique has some limitations. One limitation is the lack of validation of patients with combined iron and fat deposition in the liver, especially in high HIC (*T*_2_* < 2.0 msec) and high PDFF cases. The second limitation is that the TEs are not set at the multiple of 2.3 msec for long TE data acquisition protocol. This could result in PDFF underestimation for minor iron load patient, such as the case in Figure 5. Another minor problem is that the multiecho *T*_2_*-weighted images have to be transferred from the MR scanner to a local workstation for postprocessing, and it takes several minutes for an HIC map to be generated. Since the postprocessing on a local workstation is automatic, it is still practical. It would be ideal to generate the HIC map immediately after image acquisition on the MRI scanner. 

## 5. Conclusion

We have developed a simple, quick, and reliable method that can noninvasively measure hepatic iron overload using a standard 1.5-T MRI scanner. By combining different MEGE data acquisition strategies and an in-house image postprocessing tool, our technique cannot only measure a wide range of clinically relevant HIC values from 1.2 mg/g to greater than 45.0 mg/g dry weight, but also quantify fat/water fraction simultaneously. It generates an absolute HIC map and transfers it to the electronic medical record system, which makes it convenient for the physicians to diagnose and measure hepatic overload.
